# Glycine Betaine-Induced Responses Under Saline Stress in *Passiflora edulis* f. *flavicarpa*

**DOI:** 10.3390/plants15172592

**Published:** 2026-08-25

**Authors:** Leonardo de Almeida Oliveira, Nasratullah Habibi, Naveedullah Sediqui, Niamatullah Dawlatzai, Naoki Terada, Atsushi Sanada, Kaihei Koshio

**Affiliations:** 1 Faculty of International Agriculture and Food Studies, Tokyo University of Agriculture, Sakuragaoka 1-1-1, Setagaya-ku, Tokyo 156-8502, Japan; naweedone@gmail.com (N.S.); niamat.dawlatzai@gmail.com (N.D.); nt204361@nodai.ac.jp (N.T.); a3sanada@nodai.ac.jp (A.S.); koshio@nodai.ac.jp (K.K.); 2 Faculty of Agriculture, Balkh University, Balkh 1701, Afghanistan; 3 Department of Horticulture, Faculty of Agriculture, Alberoni University, Kohistan 1254, Afghanistan

**Keywords:** glycine betaine, salinity stress, *Passiflora edulis*, metabolomics, GC–MS, leaf water potential, osmotic adjustment, tropical fruit crops

## Abstract

Salinity constrains tropical fruit production, yet the physiological and metabolic responses of *Passiflora edulis* Sims f. *flavicarpa* to salt stress and glycine betaine (GB) remain insufficiently characterized. Passion fruit seedlings were evaluated in a completely randomized 2 × 3 factorial design with two GB treatments (0 and 100 mM), three salinity levels (0, 1.6, and 3.2 dS m^−1^), and five biological replicates per combination. Two-way ANOVA followed by Tukey’s test at 5% was used where applicable. Leaf water potential, SPAD, detached-leaf water loss, and GC–MS-based metabolite profiles were assessed. At 3.2 dS m^−1^, plants without GB exhibited the most negative leaf water potential, whereas GB maintained a significantly less negative value. SPAD remained relatively stable, while detached-leaf water-loss patterns varied among treatments. Metabolomic profiling revealed changes in sugars, amino acids, and organic acids. Under the higher salinity treatment, GB was associated with higher levels of glucose, sucrose, glycine, proline, citric acid, malic acid, and succinic acid, together with a distinct PCA profile. These findings indicate that GB partially alleviated effects of the higher salinity treatment by improving plant water status and modifying primary metabolism.

## 1. Introduction

Salinity is a major environmental constraint to agricultural productivity, particularly in irrigated and semi-arid production systems where insufficient rainfall, high evapotranspiration, poor drainage, and repeated irrigation can promote salt accumulation in soils. Plant exposure to salinity initially produces osmotic stress by decreasing the water potential of the root environment and restricting water uptake. Prolonged exposure may additionally cause ionic stress through the accumulation of sodium (Na^+^) and chloride (Cl^−^) in plant tissues [1]. Excessive Na^+^ disrupts potassium (K^+^) acquisition and cellular K^+^ homeostasis, affecting enzyme activity, membrane transport, stomatal regulation, and other processes required for growth [2]. Salinity also alters photosynthetic activity and promotes the excessive production of reactive oxygen species (ROS), which can damage membranes, proteins, pigments, and nucleic acids when antioxidant defenses are insufficient [3,4,5].

Yellow passion fruit, *P. edulis* f. *flavicarpa*, is an economically important tropical fruit crop, but its production is frequently concentrated in regions where soil and irrigation-water salinity can limit plant establishment and productivity. Studies comparing cultivated and wild *Passiflora* species have demonstrated that salinity can reduce stomatal conductance, transpiration, photosynthetic rate, chlorophyll content, leaf osmotic potential, nutrient acquisition, and plant biomass, with cultivated *P. edulis* f. *flavicarpa* generally showing greater sensitivity than some wild relatives [6]. Long-term irrigation with moderately to highly saline water has also been associated with Na^+^ and Cl^−^ accumulation, reduced shoot biomass, changes in antioxidant capacity, and the regulation of stress-responsive genes in yellow passion fruit [7]. The effects of salinity may vary according to developmental stage, as salt exposure during vegetative and flowering periods can impair plant water status, gas exchange, mineral nutrition, and fruit production [8]. At earlier developmental stages, increasing salinity reduces seed germination and seedling vigor and increases hydrogen peroxide accumulation in salt-sensitive *Passiflora* genotypes [9]. Collectively, these studies establish the sensitivity of passion fruit to salinity but also demonstrate considerable variation according to genotype, developmental stage, stress intensity, and exposure duration.

Plants respond to osmotic and ionic stress through stomatal regulation, ion compartmentalization, antioxidant defense, and the accumulation of compatible solutes. Among these compounds, glycine betaine (GB) has received considerable attention because it can accumulate without severely interfering with cellular metabolism and may contribute to osmotic adjustment, macromolecular stabilization, and protection of photosynthetic structures [10,11]. GB has also been associated with the maintenance of membrane integrity, regulation of ion balance, enhancement of antioxidant activity, and modulation of stress-responsive signaling pathways [11,12,13,14]. These functions make the exogenous application of GB a potentially useful strategy for improving plant performance under environmental stress.

Nevertheless, the mechanisms underlying GB-mediated protection remain incompletely resolved. GB has traditionally been described primarily as an osmoprotectant that directly supports cellular hydration and stabilizes proteins and membranes. More recent evidence suggests that its effects may also involve broader regulation of ion transport, phytohormone signaling, gene expression, antioxidant metabolism, and central metabolic pathways [12,13,14]. Moreover, the effectiveness of exogenous GB is influenced by plant species, endogenous GB accumulation capacity, applied concentration, developmental stage, application method, and stress severity [13,14]. Therefore, GB application should not necessarily be expected to restore all physiological characteristics uniformly. Instead, it may promote specific combinations of physiological and metabolic adjustments that improve acclimation under particular stress conditions.

Maintenance of plant water status and photosynthetic function is central to survival under salinity. Salt-induced reductions in stomatal conductance and carbon assimilation restrict photosynthetic carbon gain, while ion toxicity and oxidative stress may further impair the photosynthetic apparatus [15]. Soluble sugars can contribute to osmotic adjustment, membrane protection, ROS regulation, and stress signaling, whereas amino acids and organic acids connect osmotic regulation with nitrogen assimilation, redox balance, and energy production [16,17]. Changes in these metabolite pools may therefore complement physiological measurements such as chlorophyll concentration or water loss.

Metabolomics provides a system-level approach for characterizing these biochemical responses. Gas chromatography–mass spectrometry (GC–MS) is particularly suitable for detecting changes in primary metabolites, including sugars, amino acids, sugar alcohols, and organic acids. Previous metabolomic studies have demonstrated that abiotic stress can substantially reorganize central carbon and nitrogen metabolism and that the resulting metabolic profiles may distinguish stress intensities, tolerant and sensitive genotypes, and treatment responses [18,19,20]. Recent integrative research combining physiological measurements, transcriptomics, and metabolomics has further shown that exogenous GB can modify antioxidant activity, osmotic regulation, energy metabolism, and stress-responsive pathways under salinity [21]. Nevertheless, integrated physiological and metabolomic investigations of GB-mediated salinity responses in perennial tropical fruit crops remain limited.

Recent studies in yellow passion fruit have shown that exogenous GB can modify plant water relations and physiological responses under drought stress and induce distinct primary metabolic configurations under heat and cold stress [22,23]. These findings indicate that GB responses in *P. edulis* f. *flavicarpa* extend beyond passive osmotic protection and may involve stress-specific metabolic adjustment. However, the combined physiological and metabolic responses of *P. edulis* f. *flavicarpa* to exogenous GB under salinity have not yet been systematically characterized, representing a specific gap in our understanding of GB-mediated salt-stress responses in this species.

Therefore, this study aimed to evaluate the physiological and metabolic responses of *P. edulis* f. *flavicarpa* seedlings to increasing salinity and to determine whether the effects of exogenous GB depended on stress intensity. By combining leaf water potential, SPAD values, detached-leaf water loss, and GC–MS-based metabolite profiling, the study aimed to characterize how GB application was associated with plant water status and treatment-dependent differences in primary metabolite concentrations and overall metabolic profiles under salinity. The principal findings showed that GB-associated responses were strongest under higher salinity, where treated plants maintained higher leaf water potential and exhibited coordinated changes in soluble sugars, amino acids, organic acids, and GABA-related compounds, indicating a stress-intensity-dependent role in physiological protection and metabolic acclimation.

## 2. Results

### 2.1. Leaf Water Potential

In the Results, Discussion, and figures, several abbreviations are used: “S0” refers to control, with no NaCl added, “S1” indicates lower salinity stress, and “S2” denotes higher salinity stress. “GB0” corresponds to no glycine betaine application, whereas “GB1” signifies glycine betaine application.

**Figure 1:**
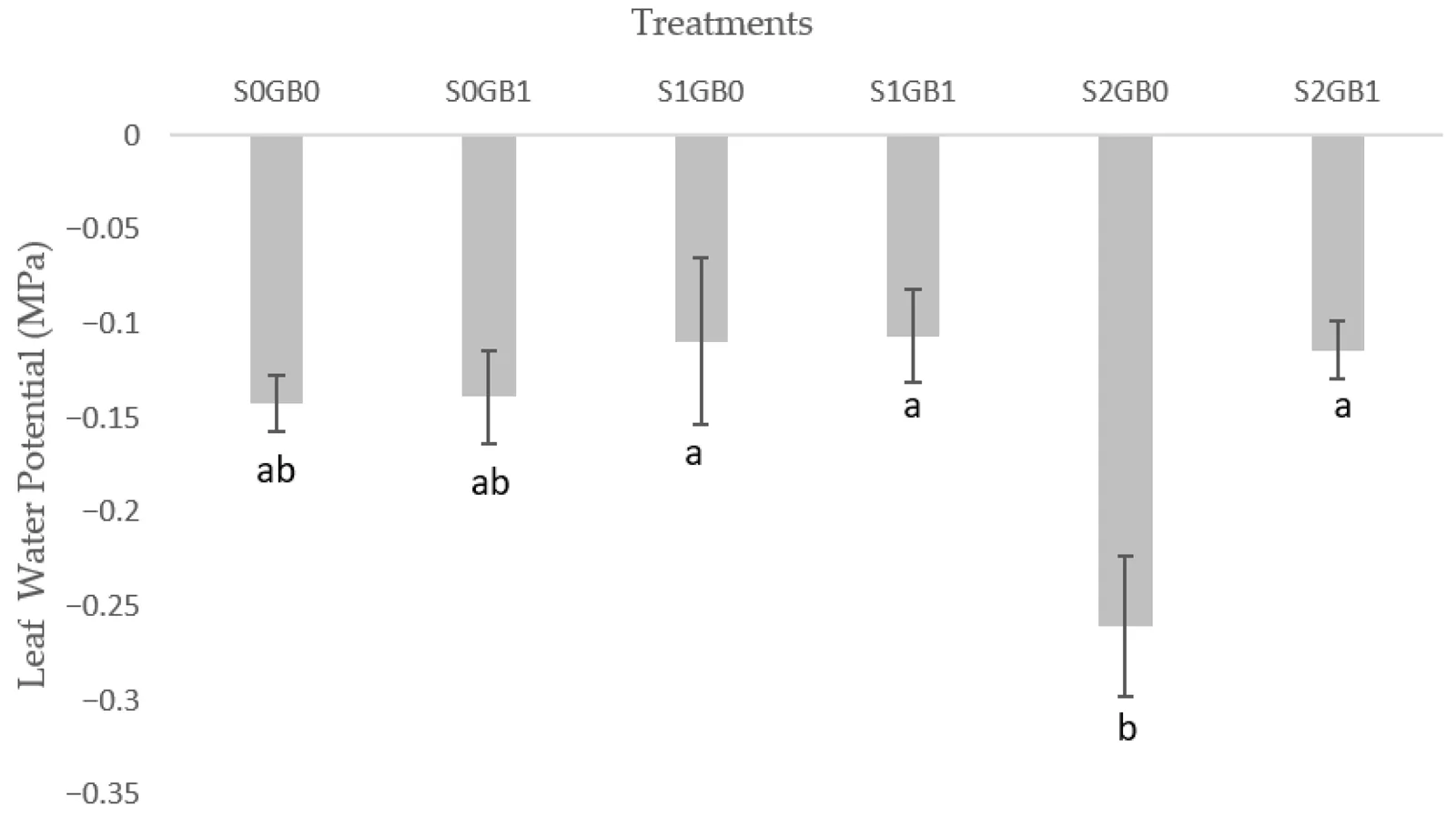
Leaf water potential of passion fruit seedlings under salinity and glycine betaine treatments. “S0” represents control salinity (no NaCl added), “S1” indicates lower salinity (1.6 dS m^−1^), and “S2” denotes higher salinity (3.2 dS m^−1^). “GB0” corresponds to no glycine betaine application, whereas “GB1” signifies glycine betaine application. Means sharing at least one lowercase letter are not significantly different according to Tukey’s test at the 5% probability level.

Leaf water potential differed among salinity and glycine betaine treatments (Figure 1). Under control conditions, GB-treated and untreated plants showed similar values. The lower-salinity treatments had numerically less negative leaf water potential values than the controls, but did not differ significantly from the controls or from each other.

Under higher salinity, S2GB0 exhibited the most negative leaf water potential. It differed significantly from S1GB0, S1GB1, and S2GB1 but remained statistically similar to the control treatments. In contrast, S2GB1 maintained a significantly less negative value than S2GB0 and remained statistically similar to the control and lower-salinity treatments, indicating that GB partially mitigated the reduction in leaf water potential under higher salinity.

### 2.2. SPAD Index

**Figure 2:**
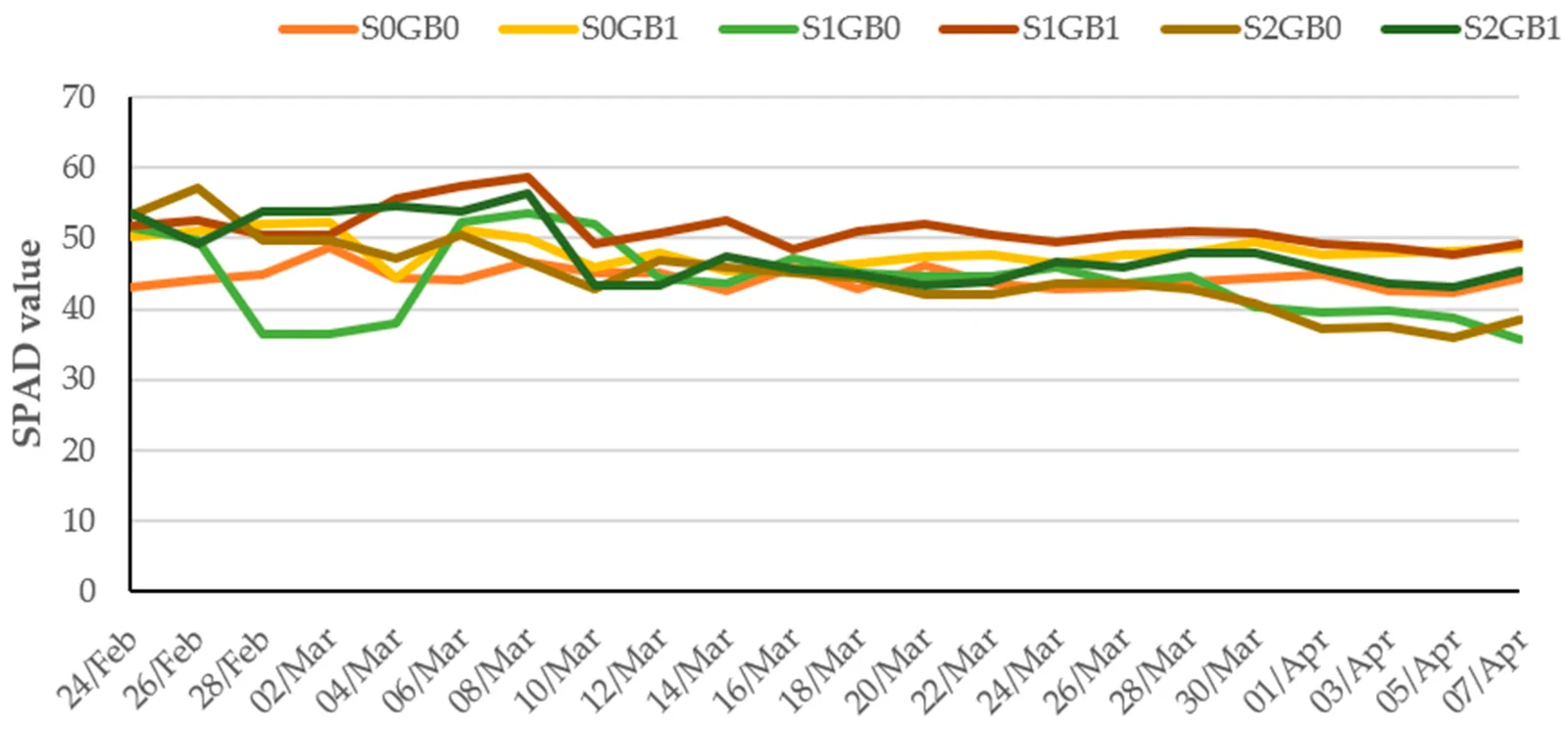
SPAD values of passion fruit seedlings under salinity and glycine betaine treatments. “S0” represents control salinity (no NaCl added), “S1” indicates lower salinity (1.6 dS m^−1^), and “S2” denotes higher salinity (3.2 dS m^−1^). “GB0” corresponds to no glycine betaine application, whereas “GB1” signifies glycine betaine application.

SPAD values generally remained within a range of approximately 40–55 units throughout the 45-day experimental period, although treatment-specific patterns became more evident toward the end of the experiment (Figure 2). Under control conditions, S0GB0 and S0GB1 showed relatively similar values and minor temporal fluctuations, without a clear difference between the two treatments.

Under lower salinity, S1GB0 and S1GB1 initially followed broadly similar patterns. However, toward the end of the experiment, SPAD values in S1GB0 were lower than those in S1GB1 and continued to decline, whereas S1GB1 appeared to stabilize. Under higher salinity, a similar response was observed: S2GB0 ended the experiment with lower SPAD values and a continuing downward trend, while S2GB1 remained comparatively stable.

### 2.3. Detached-Leaf Water Loss

Repeated-measures ANOVA detected significant effects of salinity (*p* = 0.0034) and time (*p* < 0.001) on detached-leaf water loss. In contrast, the overall effect of GB was not significant (*p* = 0.158), and no significant salinity × time, GB × time, or salinity × GB × time interactions were detected (*p* > 0.05).

**Figure 3:**
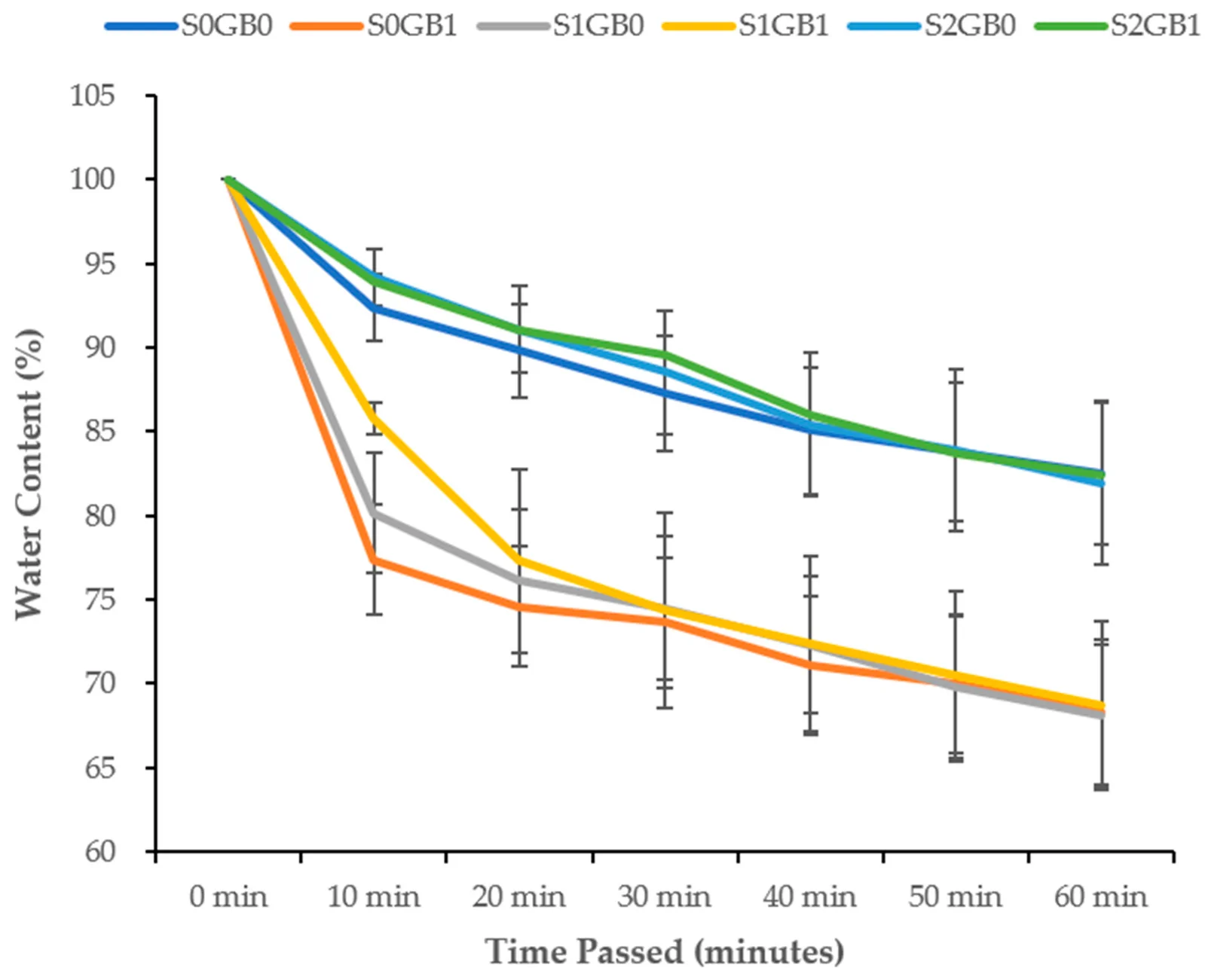
Detached-leaf water loss patterns of passion fruit seedlings under salinity and glycine betaine treatments. “S0” represents control salinity (no NaCl added), “S1” indicates lower salinity (1.6 dS m^−1^), and “S2” denotes higher salinity (3.2 dS m^−1^). “GB0” corresponds to no glycine betaine application, whereas “GB1” signifies glycine betaine application.

Water content decreased progressively in all detached leaves during the 60 min evaluation period, although the pattern differed among treatment combinations (Figure 3). Under control conditions, S0GB1 exhibited a faster initial decline and lower water-content values than S0GB0, indicating that GB did not improve detached-leaf water retention in the absence of salinity.

Under lower salinity, both S1GB0 and S1GB1 showed rapid initial declines and remained among the treatments with the lowest water-content values. S1GB1 initially retained slightly more water than S1GB0, but the curves converged toward the end of the evaluation. Under higher salinity, S2GB0 and S2GB1 followed nearly overlapping patterns, with comparatively gradual declines and higher water-content values throughout the 60 min period. Ultimately, GB did not produce a consistent improvement in detached-leaf water loss, and its effect varied according to salinity level. The water-loss trajectories described above were evaluated descriptively. At 60 min, two-way ANOVA detected no significant effects of salinity, GB application, or their interaction on final detached-leaf water content (*p* > 0.05).

### 2.4. Metabolite Concentrations

Metabolite concentrations showed treatment-dependent variation, with the clearest statistical separation frequently involving S2GB1 (Table 1). Among the carbohydrates, glucose and sucrose were significantly higher in S2GB1 than in all other treatments. In plants without GB application, sucrose decreased numerically as salinity increased. S2GB0 had a significantly lower sucrose concentration than the two control treatments and S1GB0, although it did not differ significantly from S1GB1. Fructose showed a different response, as S1GB0 and S1GB1 had significantly lower fructose concentrations than S0GB0, but did not differ significantly from S0GB1, S2GB0, or S2GB1.

Proline concentration was significantly higher in S2GB1 than in all other treatments. For glycine, S2GB1 was significantly higher than S1GB0, S1GB1, and S2GB0, but did not differ significantly from the control treatments. Glutamic acid showed limited statistical separation, with a significant difference detected only between S0GB1 and S1GB1. GABA concentrations were significantly lower in S1GB0, S1GB1, and S2GB0 than in the control treatments and S2GB1. Consequently, GABA in S2GB1 remained statistically similar to the control treatments.

Malic acid concentrations in S0GB0, S0GB1, and S2GB1 were statistically similar and significantly higher than those in S1GB0, S1GB1, and S2GB0. Citric acid was significantly higher in S2GB1 than in all other treatments. Among the remaining treatments, S0GB0 was significantly higher than S1GB1, whereas the intermediate treatments had overlapping statistical groupings. Succinic acid was also significantly higher in S2GB1 than in all other treatments. S0GB1 and S2GB0 had significantly higher succinic acid concentrations than S1GB0 and S1GB1, while S0GB0 did not differ significantly from either group. Overall, the most pronounced metabolite differences associated with GB application occurred at the higher salinity level, particularly between S2GB1 and S2GB0.

### 2.5. Principal Component Analysis

**Figure 4:**
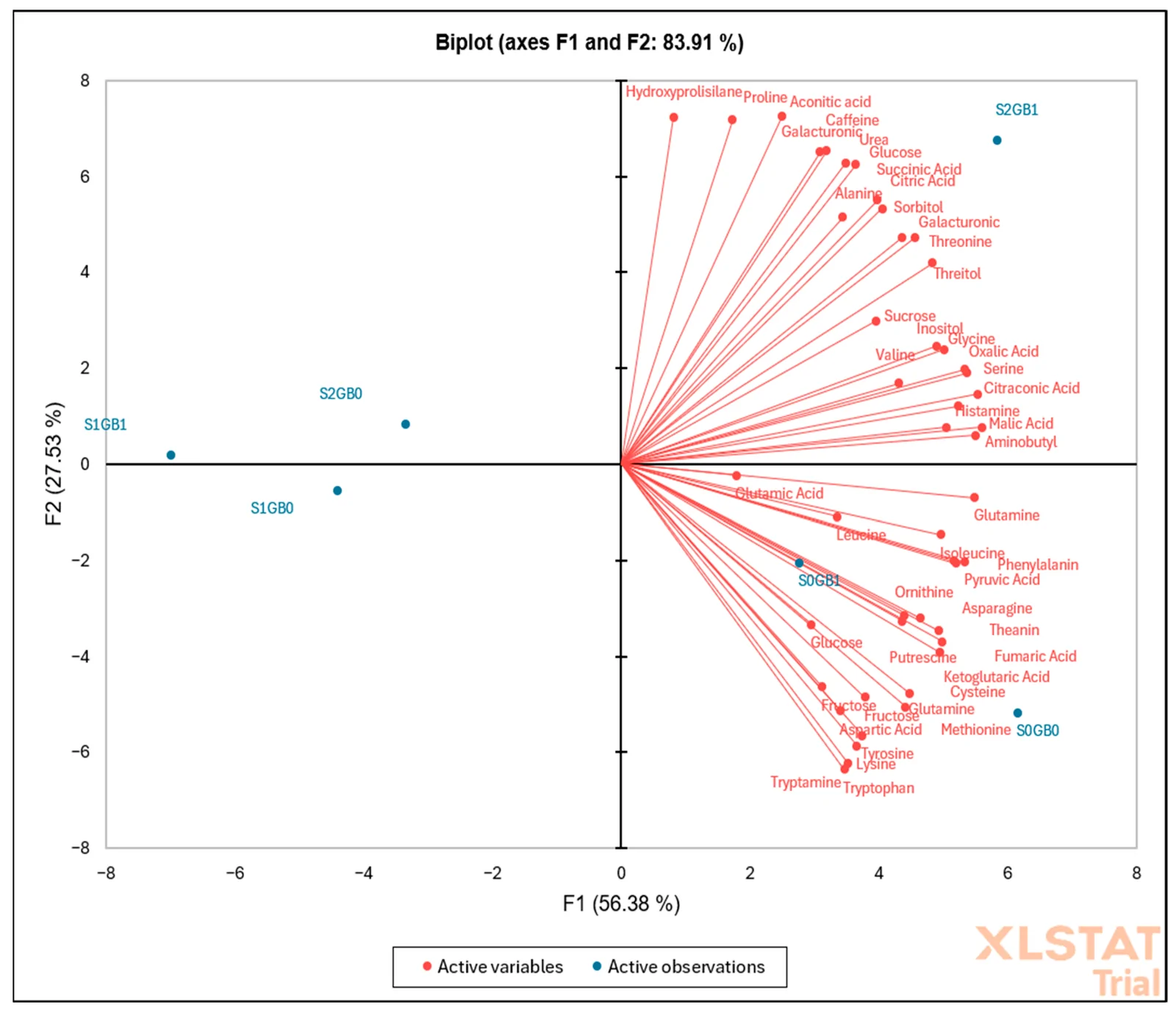
Principal component analysis biplot of primary metabolite profiles in passion fruit seedlings under salinity and glycine betaine treatments. “S0” represents control salinity (no NaCl added), “S1” indicates lower salinity (1.6 dS m^−1^), and “S2” denotes higher salinity (3.2 dS m^−1^). “GB0” corresponds to no glycine betaine application, whereas “GB1” signifies glycine betaine application. Repeated metabolite names represent distinct chromatographic peaks detected at different retention times and retained as separate variables in the PCA.

Principal component analysis (PCA) revealed clear differences in metabolite profiles among the salinity and glycine betaine treatments (Figure 4). The first two principal components explained 83.91% of the total variance, with PC1 accounting for 56.38% and PC2 accounting for 27.53%.

S1GB0, S1GB1, and S2GB0 were positioned on the negative side of PC1, whereas S0GB0, S0GB1, and S2GB1 were located on the positive side. S2GB1 was clearly separated in the upper-right region of the biplot and was associated with proline, aconitic acid, galacturonic acid, glucose, succinic acid, citric acid, sorbitol, threonine, and threitol. In contrast, S0GB0 was positioned in the lower-right region and was associated with methionine, tryptophan, tryptamine, lysine, fructose, pyruvic acid, phenylalanine, and fumaric acid. The distinct position of S2GB1 relative to S2GB0 indicates that GB application under higher salinity was associated with a different overall metabolic profile, particularly involving carbohydrates, amino acids, sugar alcohols, and organic acids.

### 2.6. Z-Score Heatmap

**Figure 5:**
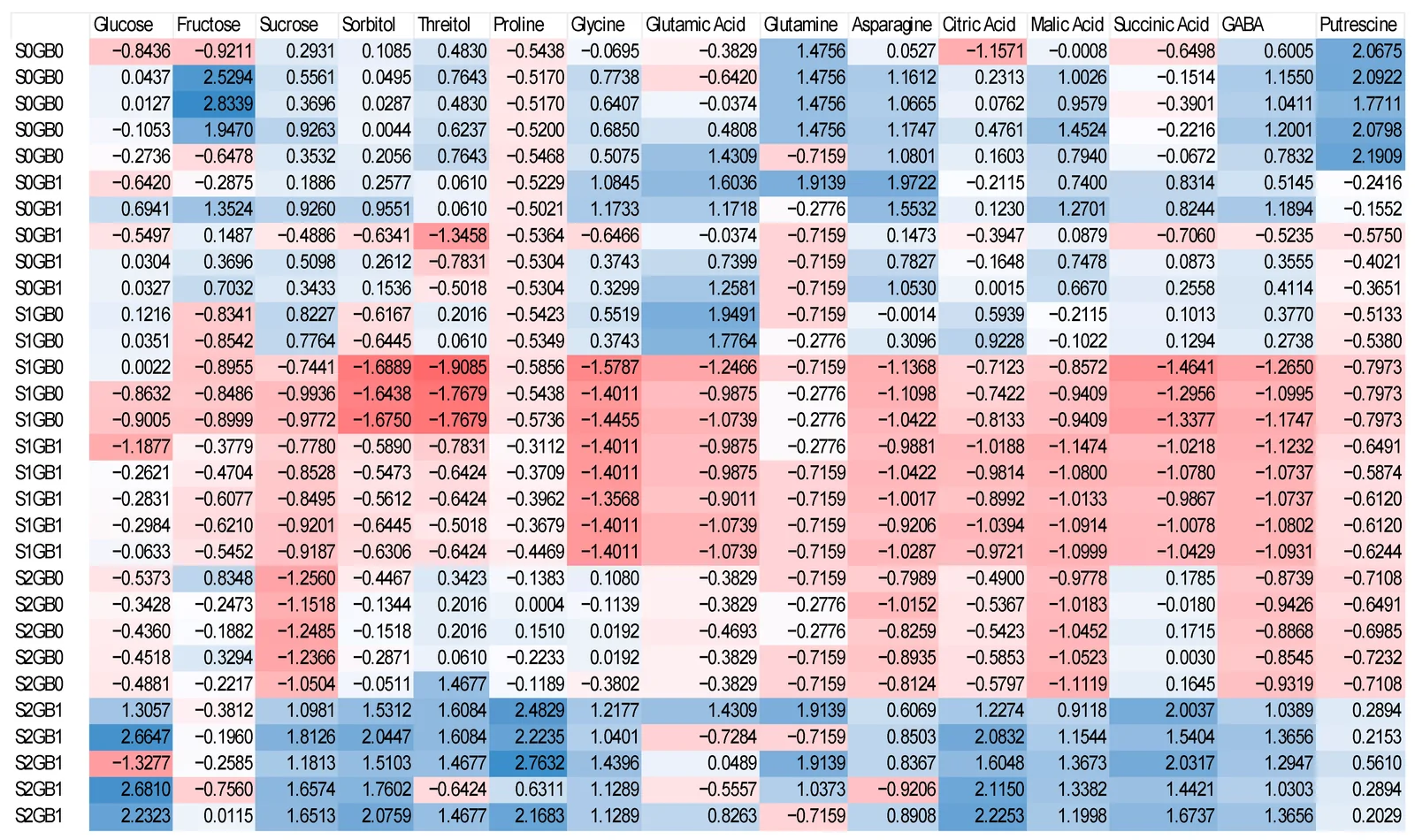
Heatmap showing Z-score normalized metabolite profiles under salinity stress conditions. “S0” represents control salinity (no NaCl added), “S1” indicates lower salinity (1.6 dS m^−1^), and “S2” denotes higher salinity (3.2 dS m^−1^). “GB0” corresponds to no glycine betaine application, whereas “GB1” signifies glycine betaine application. Blue indicates positive Z-scores (above-average relative abundance for a given metabolite), whereas red indicates negative Z-scores (below-average relative abundance); darker shades indicate greater absolute deviations from the mean, while lighter shades indicate values closer to the mean.

The Z-score heatmap revealed distinct treatment-dependent patterns in the relative abundance of the selected metabolites under salinity stress (Figure 5). Biological replicates within each treatment generally exhibited similar metabolic profiles, indicating consistency among biological replicates. In contrast, clear differences were observed among treatment groups, reflecting the effects of both salinity level and glycine betaine application on metabolite accumulation.

Lower salinity without glycine betaine (S1GB0) was characterized by a predominance of metabolites with below-average relative abundance, whereas higher salinity combined with glycine betaine (S2GB1) showed increased relative abundance for several metabolites. Changes were particularly evident among metabolites associated with carbohydrate metabolism (glucose and sucrose), amino acid metabolism (proline and glutamine), and the tricarboxylic acid (TCA) cycle (citric acid, malic acid, and succinic acid). Ultimately, the heatmap therefore showed that salinity and glycine betaine application were associated with distinct relative-abundance patterns among the selected metabolites.

## 3. Discussion

### 3.1. Leaf Water Potential

S2GB1 maintained a significantly less negative leaf water potential, demonstrating that exogenous GB partially improved plant water status under higher salinity. Protective effects of GB on plant water relations have also been reported in other species and have been associated with osmotic adjustment, membrane stabilization, and maintenance of cellular hydration under abiotic stress [12,24,25].

Under lower salinity, leaf water potential was statistically similar to control conditions, and GB-treated and untreated plants did not differ. Metabolic and osmotic adjustments may have buffered lower salinity stress through reorganization of central metabolism and activation of protective mechanisms [3,20]. The clearer response under higher salinity indicates that the effect of GB depended on stress intensity.

Salinity lowers external water potential and restricts root water uptake, producing physiological drought even when water remains in the substrate [26]. Prolonged salinity may also cause ionic stress through Na^+^ and Cl^−^ accumulation [27,28]. However, tissue ion concentrations were not measured, so their contribution to the strongly negative leaf water potential in S2GB0 cannot be determined.

The less negative leaf water potential of S2GB1 may be associated with several reported functions of GB. As a compatible solute, GB can support osmotic adjustment and cellular hydration without substantially disrupting metabolism [14,24]. It has also been associated with membrane and protein stabilization, aquaporin and ion-transport regulation [13], and antioxidant defense under salinity [3,29]. However, these mechanisms were not measured here.

Stomatal closure can limit water loss under osmotic stress but may also restrict CO_2_ assimilation [30]. GB has been associated with physiological regulation and photosynthetic performance, potentially through osmotic balance [25]. Because stomatal conductance was not measured, its contribution cannot be determined. The leaf water potential data nevertheless show that GB partially improved water status under higher salinity.

A further limitation is that leaf water potential was measured only once, at the end of the 45-day experimental period. Therefore, these measurements represent endpoint differences in plant water status and do not capture the temporal dynamics of water-potential adjustment or osmotic acclimation during salinity exposure. Repeated measurements throughout the experimental period would be required to characterize these responses over time.

### 3.2. SPAD Index

SPAD values remained within a relatively narrow range throughout most of the experimental period, indicating that salinity did not cause an immediate or pronounced decline in relative chlorophyll content. Relative chlorophyll and photosynthetic stability during stress have been reported in plants capable of maintaining effective photochemical, osmotic, and antioxidant protection [3,29,31]. However, treatment-specific differences became more evident toward the end of the experiment, indicating that the response was not completely uniform across salinity and GB treatments.

Under control conditions, S0GB0 and S0GB1 showed similar SPAD values and only minor temporal fluctuations. This limited response to GB in the absence of salinity is consistent with the view that its protective effects are generally more evident under environmental stress than under favorable conditions [13,24].

Under lower salinity, S1GB0 and S1GB1 initially followed similar patterns, but S1GB0 declined near the end whereas S1GB1 appeared to stabilize. Salinity can affect chlorophyll metabolism through oxidative and nutritional disturbances [3,26], while osmotic and antioxidant protection may preserve thylakoid membranes and pigment–protein complexes [20,28]. However, these late-stage differences were evaluated descriptively and not subjected to inferential analysis.

Under higher salinity, S2GB0 also declined late in the experiment while S2GB1 remained comparatively stable. Higher salinity can disrupt chloroplast organization and increase ROS production [3,4,5], potentially damaging chlorophyll and photosystem components [32]. GB has been associated with membrane stabilization, osmotic adjustment, antioxidant protection, and maintenance of photosystem II under stress [12,14,25,29]. However, ROS, MDA, and antioxidant enzyme activities were not measured, so oxidative-stress mitigation cannot be confirmed.

Nevertheless, SPAD measurements estimate relative chlorophyll content rather than photosynthetic activity directly. Photosynthetic performance may decline under salinity even when SPAD values remain relatively stable because of stomatal limitations, reduced CO_2_ assimilation, and inhibition of carbon-fixation processes [15,30]. Temporal fluctuations may also reflect changes in carbon allocation, nitrogen metabolism, pigment turnover, and the availability of nitrogen and magnesium required for chlorophyll biosynthesis [20,28]. Therefore, the maintenance of SPAD values should be interpreted as relative preservation of chlorophyll rather than evidence that photosynthetic function was fully maintained. The results suggest that GB was associated with improved late-stage chlorophyll stability under lower and higher salinity, but direct measurements of gas exchange and photosynthetic efficiency would be required to confirm functional protection.

### 3.3. Detached-Leaf Water Loss

Detached-leaf water content decreased throughout the 60 min evaluation period in all treatments, but the response did not follow a simple progression with increasing salinity. S0GB0, S2GB0, and S2GB1 maintained comparatively higher water-content values, whereas S0GB1, S1GB0, and S1GB1 showed faster declines and lower final values. These patterns indicate that the effect of GB on detached-leaf water loss depended on salinity level and was not consistently beneficial.

Under control conditions, S0GB1 declined faster than S0GB0, indicating that GB did not improve water retention without salinity, consistent with stress-dependent GB effects [12,13]. Under lower salinity, S1GB0 and S1GB1 both declined rapidly; S1GB1 initially retained slightly more water, but the curves later converged. Salinity can alter water relations through lower external water potential and changes in membrane and hydraulic regulation [1,2,3], although the assay cannot identify the responsible mechanisms.

The absence of a clear GB benefit under lower salinity contrasts with reports that exogenous GB can support osmotic adjustment, membrane stabilization, and reduced membrane leakage under abiotic stress [10,12,14,24,33]. Improved water retention following GB application has also been reported in barley, wheat, and tomato under osmotic or saline stress [10,29]. This difference suggests that the effectiveness of GB may depend on species, developmental stage, stress intensity, application conditions, and the physiological variable evaluated.

Under higher salinity, S2GB0 and S2GB1 followed nearly overlapping curves and maintained comparatively high water content. Their slower decline may reflect acclimation before excision, including osmotic adjustment or lower transpiration [15,30], but stomatal conductance was not measured. Although GB has been associated with membrane and protein stabilization, antioxidant defense, and aquaporin-related transport [3,4,12,34], it produced no detectable additional improvement under higher salinity in this assay.

The greatest reductions in water content generally occurred during the first 10–20 min after excision, followed by a more gradual decline. This pattern likely reflects rapid water loss immediately after detachment, followed by progressive stomatal closure and an increasing relative contribution of cuticular water loss. Similar biphasic patterns have been described in detached leaves exposed to water stress [35,36]. Because stomatal and cuticular conductance were not measured separately, their respective contributions cannot be determined from the present data.

Pre-existing metabolic and structural adjustments may also have contributed. Compatible solutes support osmotic balance and cellular hydration [20,28], while soluble sugars may stabilize membranes and proteins [16,17]. Salinity-induced changes in cuticles, cell walls, and tissue organization can also influence leaf water movement [36], but these factors were not measured.

Although descriptive differences among treatment combinations were evident during the early stages of leaf dehydration, repeated-measures analysis showed that the temporal pattern of water loss was not significantly modified by GB or by its interaction with salinity. Salinity nevertheless exerted a significant overall effect on detached-leaf water content across the evaluation period.

### 3.4. Metabolite Concentrations

Salinity and glycine betaine application produced substantial changes in the primary metabolite profile of *P. edulis* f. *flavicarpa*, particularly in compounds associated with osmotic adjustment, carbon metabolism, nitrogen metabolism, and stress signaling. The most pronounced responses were generally observed in S2GB1, which had significantly higher concentrations than S2GB0 for eight of the ten metabolites summarized in Table 1. Similar reorganization of primary metabolism has been widely reported under osmotic and saline stress, where coordinated changes in metabolite pools contribute to cellular protection, energy supply, and maintenance of physiological homeostasis [19,20,28].

Glycine was numerically highest in S2GB1 and lowest in S1GB1. S2GB1 had a significantly higher glycine concentration than S1GB0, S1GB1, and S2GB0, although it remained statistically similar to the control treatments. Glycine participates in photorespiration and one-carbon metabolism and may contribute to redox and antioxidant metabolism [3,37]. Its connection with GB synthesis and turnover makes the S2GB1 response consistent with broader amino-acid adjustment [12,13], but endogenous GB metabolism was not evaluated and causality cannot be established.

Carbohydrate metabolism also showed a strong treatment-dependent response. Glucose and sucrose concentrations were significantly higher in S2GB1 than in all other treatments. Soluble sugars contribute to osmotic adjustment, cellular hydration, membrane and protein stabilization, and stress signaling [16,17,38]. Their accumulation may therefore reflect altered carbon allocation under higher salinity.

Sucrose decreased numerically with increasing salinity in plants without GB application, whereas S2GB1 had a significantly higher concentration than all other treatments. As the principal transport sugar in many plants, sucrose connects photosynthetic carbon assimilation with energy supply, growth, osmotic regulation, and stress signaling [38]. Its higher concentration under higher salinity with GB application may indicate that carbon availability or sucrose turnover differed from that in S2GB0. Sucrose may also support membrane and protein stability during dehydration [16]. Nevertheless, because photosynthetic carbon assimilation and the activities of sucrose-metabolizing enzymes were not measured, the specific process responsible for this accumulation remains uncertain.

Fructose responded differently from glucose and sucrose. Both lower-salinity treatments had significantly lower fructose concentrations than S0GB0, but they did not differ significantly from S0GB1 or either higher-salinity treatment. This non-linear response indicates that individual soluble sugars responded differently according to salinity intensity. The contrasting patterns of glucose, fructose, and sucrose emphasize that carbohydrate metabolism under stress cannot be represented by changes in a single sugar and may involve differences in synthesis, transport, interconversion, and utilization [37,39].

Proline showed the strongest amino-acid response and was significantly higher in S2GB1 than in all other treatments. Proline is associated with plant responses to water limitation [40] and may support osmotic adjustment, membrane stability, ROS regulation, and protein protection [10]. Its concentration in S2GB1 indicates an association between GB application and the proline pool, consistent with reports in other crops [24], but the underlying mechanism cannot be determined from concentration data alone.

Malate, citrate, and succinate also responded to treatment. Malate in S2GB1 was significantly higher than in the lower-salinity treatments and S2GB0, but remained statistically similar to the controls. Citrate and succinate were significantly higher in S2GB1 than in all other treatments. These TCA-cycle intermediates connect respiration, energy production, carbon allocation, pH regulation, and nitrogen metabolism [37,39]. Their response is consistent with stress-related changes in TCA-cycle intermediates [19,20,28], but metabolite concentrations do not demonstrate pathway flux or respiratory activity.

Glutamic acid showed limited treatment-dependent variation. A significant difference was detected only between S0GB1 and S1GB1, whereas all other treatments had overlapping Tukey groupings. Glutamate occupies a central position at the interface between carbon and nitrogen metabolism and serves as a precursor for several stress-related metabolites, including proline and GABA [41]. However, because the statistical separation was limited and metabolite concentrations do not provide direct information about metabolic flux, these results do not support a specific interpretation of changes in glutamate metabolism.

GABA concentrations were significantly lower in both lower-salinity treatments and in S2GB0 than in the control treatments and S2GB1. GABA is associated with cytosolic pH regulation, carbon–nitrogen balance, osmotic adjustment, and stress signaling [20,41]. Its maintenance at control-like concentrations in S2GB1 therefore distinguishes this treatment from S2GB0, but does not demonstrate activation of GABA-related metabolism or accumulation above control levels.

Overall, the metabolite response to GB depended on salinity level. Under higher salinity, S2GB1 had significantly higher concentrations of glucose, sucrose, proline, glycine, malate, citrate, succinate, and GABA than S2GB0. Fructose and glutamic acid did not differ significantly between these treatments. These concentration patterns indicate a distinct metabolic response but do not establish pathway regulation; the underlying mechanisms require further study.

### 3.5. Principal Component Analysis

The principal component analysis revealed clear differences among the metabolic profiles of the salinity and glycine betaine treatments. PC1 and PC2 together explained 83.91% of the total variance, indicating that the first two components represented most of the variation in the metabolite dataset. This treatment discrimination is consistent with metabolomic studies showing that abiotic stress produces coordinated changes across multiple metabolic pathways rather than isolated changes in individual compounds [18,20,28].

S1GB0, S1GB1, and S2GB0 occurred on negative PC1, whereas S0GB0, S0GB1, and S2GB1 occurred on positive PC1. Thus, the distribution was not a uniform salinity gradient: S2GB0 was closer to lower-salinity groups, while S2GB1 was clearly distinct. This separation indicates a strong association between GB and the metabolic response under higher salinity, when osmotic limitation, ionic imbalance, and oxidative stress can affect primary metabolism [3,26].

S2GB1 was associated with metabolites belonging to several functional groups, including soluble sugars, compatible solutes, amino acids, sugar alcohols, and organic acids. Glucose, proline, sorbitol, threitol, galacturonic acid, aconitic acid, citric acid, and succinic acid contributed to its distinct position in the PCA. The association of soluble sugars and sugar alcohols with S2GB1 is consistent with their reported roles in osmotic adjustment, cellular signaling, and stabilization of proteins and membranes during stress [16,38]. However, the PCA demonstrates association rather than a change in pathway activity or carbon allocation.

Proline also contributed strongly to S2GB1 separation, consistent with its roles in osmotic adjustment, ROS regulation, and membrane and protein protection [10,40]. This agrees with the concentration results and with previous reports of relationships between exogenous GB and proline accumulation [24].

Aconitate, citrate, malate, and succinate also contributed to treatment separation. These TCA-cycle intermediates connect respiration, energy production, carbon allocation, and nitrogen metabolism [37,39], and commonly change during abiotic-stress adjustment [19,28]. Their association with S2GB1 suggests reorganization of central carbon metabolism but cannot demonstrate increased respiratory flux.

The distribution of amino acids such as alanine, valine, serine, leucine, isoleucine, glutamine, and asparagine suggests that nitrogen metabolism also contributed to the separation among treatments. Abiotic stress can alter amino acid pools through changes in protein turnover, osmotic regulation, and carbon–nitrogen balance [17]. Branched-chain amino acids may additionally serve as alternative respiratory substrates during stress [19,28]. The contribution of GABA further indicates the involvement of GABA-related metabolism, which is associated with cytosolic pH regulation, stress signaling, and coordination between carbon and nitrogen metabolism [41].

S0GB0 and S0GB1 were close, indicating limited GB-related metabolic differences without salinity, consistent with the stress-dependent action of exogenous GB [12,13]. Lower-salinity treatments were also less displaced than S2GB1, supporting a response specific to the combination of higher salinity and GB [20,28].

Overall, the clustering of sugars, amino acids, sugar alcohols, and organic acids near S2GB1 shows coordinated contributions from carbon metabolism, nitrogen metabolism, osmotic regulation, and stress defense [3,16]. PCA therefore supports an association between GB application and broader metabolic differences under higher salinity, without demonstrating direct pathway regulation.

### 3.6. Z-Score Heatmap

The Z-score heatmap revealed distinct treatment-dependent patterns in the selected metabolites. The control treatments generally exhibited relative abundances closer to the dataset mean, whereas lower salinity, particularly S1GB0, was associated with below-average abundances of several metabolites. In contrast, S2GB1 showed higher relative abundances of numerous compounds, including soluble sugars, amino acids, and TCA-cycle intermediates. This pattern indicates that GB application under higher salinity was associated with a metabolic configuration that differed from both untreated salt-stressed plants and the controls.

Metabolomic reprogramming has also been observed in rice exposed to salinity, where NaCl treatment altered sugars, amino acids, polyols, and organic acids and produced distinct treatment- and genotype-dependent heatmap patterns [42]. The differentiation among control, lower-salinity, and higher-salinity treatments in the present study similarly demonstrates that salinity intensity influenced several components of primary metabolism simultaneously.

The higher relative abundances of carbohydrates, amino acids, and organic acids in S2GB1 were associated with a distinct metabolic profile under higher salinity. These compound classes participate in osmotic regulation, cellular protection, carbon–nitrogen balance, and energy metabolism, consistent with reported functions of GB during abiotic stress [14]. However, Z-scores represent relative abundance within this dataset and do not provide direct evidence of increased pathway activity or causal metabolic regulation by GB.

GB-associated metabolic profiles can also depend on time, as reported in Glycyrrhiza uralensis [21]. Although that study differed in duration and species, both datasets show that GB treatment was associated with coordinated metabolic differences under salinity, most clearly in S2GB1 here. A major limitation is the absence of tissue elemental analysis. Consequently, Na^+^ and Cl^−^ accumulation, K^+^ retention, Na^+^/K^+^ homeostasis, and any GB effect on ion regulation cannot be evaluated. Future studies should combine physiological and metabolomic measurements with ion analysis to distinguish osmotic and ionic responses.

**Figure 6:**
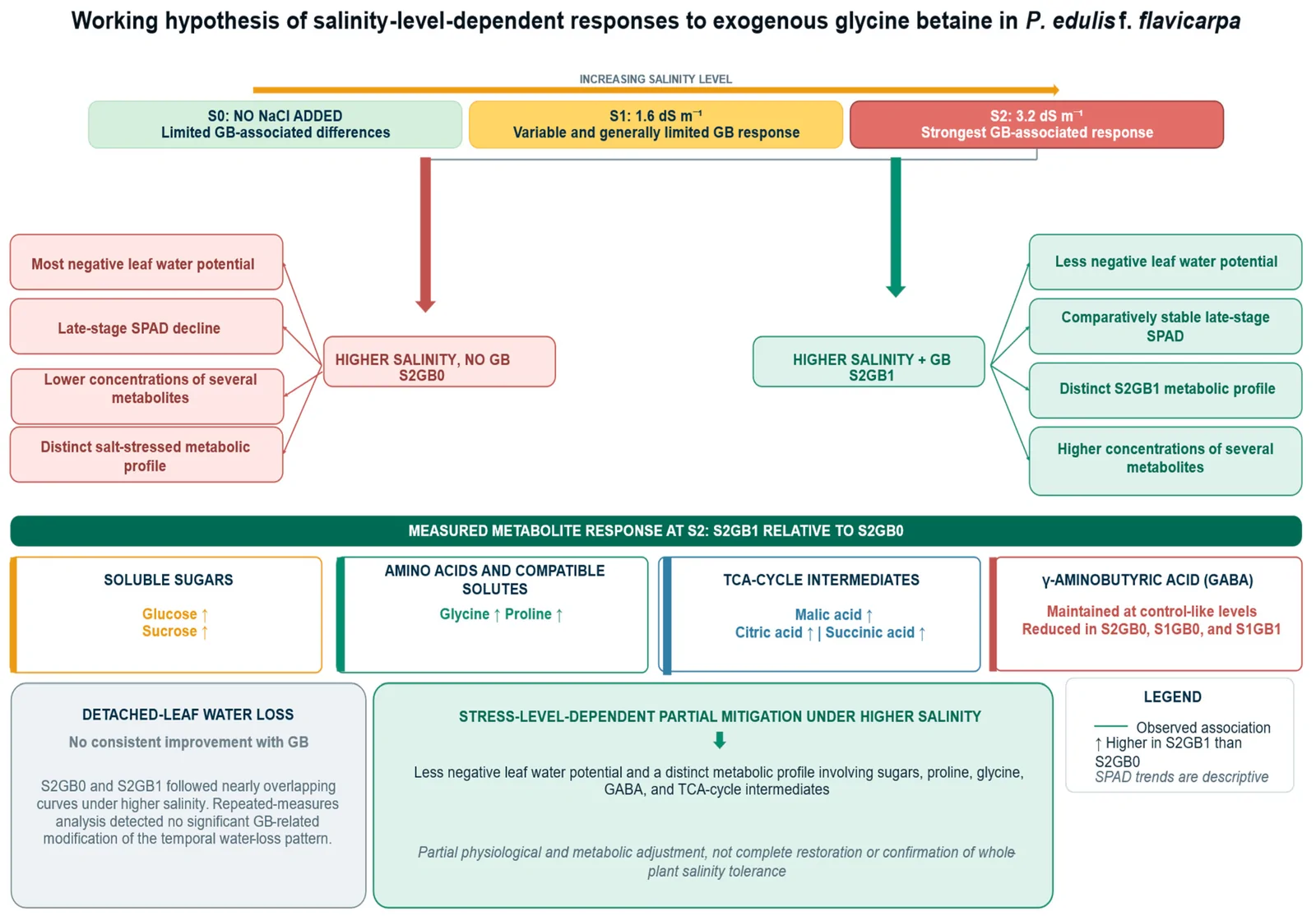
Working hypothesis summarizing the physiological and metabolic responses associated with exogenous glycine betaine application under increasing salinity in *P. edulis* f. *flavicarpa*. The proposed relationships require confirmation through ion analysis, gas-exchange measurements, and enzymatic assays.

Based on the combined physiological and metabolomic responses, a working hypothesis of GB action under salinity is presented in Figure 6. Under the higher salinity treatment, GB application was associated with less negative leaf water potential, higher concentrations of several soluble sugars, amino acids, compatible solutes, and TCA-cycle intermediates, and maintenance of GABA near control levels relative to S2GB0. However, GB did not consistently improve detached-leaf water loss. This working hypothesis therefore represents a salinity-intensity-dependent partial physiological and metabolic adjustment rather than complete restoration of salinity tolerance and requires confirmation through ion analysis, gas-exchange measurements, and enzymatic assays.

## 4. Materials and Methods

### 4.1. Experimental Design

A completely randomized design was employed in a factorial arrangement (2 × 3), consisting of two glycine betaine (GB) treatments and three salinity levels. The GB treatments consisted of a control without GB application (GB0, 0 mM) and exogenous GB application (GB1, 100 mM), with the latter concentration selected based on preliminary experiments. Salinity stress was imposed at three levels based on the electrical conductivity (EC) of the irrigation solution: No NaCl added (control), 1.6 dS m^−1^ (lower salinity), and 3.2 dS m^−1^ (higher salinity). These levels were selected based on electrical-conductivity ranges previously evaluated in *P. edulis* f. *flavicarpa* salinity studies, including 1.6 and 3.2 dS m^−1^, at which physiological and growth responses to salt stress and mitigation treatments have been investigated [43,44,45]. In the present study, they are described as the lower and higher salinity treatments solely according to their relative positions within the experimental design and not as formal irrigation-water salinity classifications.

Glycine betaine was dissolved in distilled water to prepare a 100 mM solution. Tween 20 was added as a surfactant at a rate of two drops per prepared solution. The solution was applied as a foliar spray in the evening until the leaf surfaces were uniformly wet. Because application was standardized according to leaf-surface wetness rather than a fixed solution volume, the exact volume applied per plant was not recorded. Applications were performed on days 0, 10, 20, 30, and 40 of the 45-day experimental period. Control plants received an equivalent foliar application of distilled water containing the same amount of Tween 20.

The experiment was conducted using *P. edulis* f. *flavicarpa* seedlings at the vegetative stage. Each experimental unit consisted of one individual six-month-old seedling grown in a separate 20 L pot. Five independent plants were assigned to each treatment combination, resulting in 30 experimental units. Plants were randomly assigned to treatments to minimize experimental bias.

Salinity treatments were imposed through irrigation with sodium chloride (NaCl) solutions adjusted to electrical conductivities of 1.6 and 3.2 dS m^−1^. Glycine betaine was applied as a foliar spray at a concentration of 100 mM at 10-day intervals throughout the experimental period. Salinity treatments were prepared by dissolving sodium chloride (NaCl) in local tap water to obtain irrigation solutions with electrical conductivities of 1.6 and 3.2 dS m^−1^. The electrical conductivity of each solution was verified with an EC meter (HORIBA Advanced Techno Co., Ltd., Kyoto, Japan) before every irrigation event. Control plants received the same tap water without added NaCl. The target salinity levels were applied at full concentration from the beginning of the experimental period and maintained throughout the 45-day experiment. The electrical conductivity of the substrate and drainage water was not monitored during the experiment; therefore, temporal changes in root-zone salinity could not be quantified.

Irrigation was performed whenever the tensiometer (DIK-8333 pF meter/tensiometer (Daiki Rika Kogyo Co., Ltd., Kounosu, Saitama, Japan)) reading entered the yellow or red moisture range. Each pot received sufficient solution to produce slight drainage from the bottom of the container, thereby reducing excessive salt accumulation in the substrate. Saline plants received their corresponding NaCl solution at every irrigation event, whereas control plants received an equivalent amount of non-saline irrigation water.

Prior to the main experiment, preliminary screening trials were conducted to determine an appropriate glycine betaine concentration and application frequency for *P. edulis* f. *flavicarpa*. In the first trial, eight GB concentrations (0, 2, 5, 10, 20, 50, 100, and 200 mM) were evaluated in 3-month-old seedlings subjected to stress conditions. Treatments were assessed primarily based on visible stress symptoms and SPAD chlorophyll readings to evaluate stress mitigation and potential phytotoxicity. Lower concentrations showed limited visible stress mitigation, whereas 200 mM caused mild chlorosis. The 100 mM treatment maintained SPAD values and reduced visible stress symptoms without apparent toxicity. In the second trial, weekly, biweekly, monthly, and single applications were evaluated in 1-year-old plants. Based on both preliminary trials, 100 mM GB applied at 10-day intervals was selected for the main experiment.

### 4.2. Experimental Indexes

The physiological variables included leaf water potential and SPAD values. SPAD values were measured throughout the 45-day experimental period, excluding the first three days. In addition, the detached-leaf water loss was determined along with the quantification of 53 metabolites analyzed by gas chromatography–mass spectrometry (GC–MS).

SPAD values were obtained using a chlorophyll meter (SPAD-502, Konica Minolta, Tokyo, Japan) as a non-destructive indicator of leaf chlorophyll content. This parameter is widely used to assess plant physiological status and relative chlorophyll concentration. However, SPAD readings do not directly measure photosynthetic rate.

### 4.3. Experiment Management

The experiment was conducted using seed-derived *P. edulis* f. *flavicarpa* seedlings at the vegetative stage. Because yellow passion fruit is highly heterozygous, genetic variation among seed-derived individuals could not be eliminated. Seedlings with comparable size and phenological development were selected before treatment allocation. Each experimental unit consisted of one individual six-month-old seedling grown in a separate 20 L pot. Five independent plants were assigned to each treatment combination, resulting in 30 experimental units. Plants were randomly assigned to the treatment combinations to distribute biological variability and reduce systematic experimental bias. Nevertheless, residual genetic variation among seedlings is recognized as a limitation of the experimental material.

The pots were filled with commercial Akadama soil, a granular volcanic clay substrate characterized by a slightly acidic pH of 5.5–6.5, low electrical conductivity below 0.5 dS m^−1^, total porosity of approximately 50–65%, moderate water-holding capacity of 30–45% (*v*/*v*), and minimal organic matter content below 5%. All plants were cultivated using the same substrate batch to ensure uniform growth conditions among treatments.

The experiment was conducted in a greenhouse at Tokyo University of Agriculture, Tokyo, Japan, during the winter-to-spring period. The greenhouse heating system was set to approximately 25 °C. Spot measurements recorded during the experimental period averaged 25.6 ± 6.5 °C. Nutrient management consisted of foliar fertilization with a solution prepared by dissolving 8 g of OAT House Fertilizer No. 1 and 6 g of OAT House Fertilizer No. 2 in 2 L of water. At each fertilization event, the resulting solution was distributed uniformly over the foliage of all plants rather than applied to the substrate or dosed per pot through irrigation. Foliar fertilization was performed three times per week, beginning one month after seedling establishment and continuing throughout the 45-day experimental period. According to the manufacturer, OAT House Fertilizer No. 1 contained 10.0% total nitrogen, 8.0% water-soluble P_2_O_5_, 27.0% water-soluble K_2_O, 4.0% water-soluble MgO, 0.10% water-soluble MnO, 0.10% water-soluble B_2_O_3_, 0.18% Fe, 0.002% Cu, 0.006% Zn, and 0.002% Mo, whereas OAT House Fertilizer No. 2 contained 11.0% nitrate nitrogen and 16.4% calcium.

### 4.4. Experiment Measurement

Leaf water potential was measured once at the end of the 45-day experimental period using a pressure chamber (Model 1505D, PMS Instrument Company, Albany, OR, USA). Measurements were performed in the morning at approximately 08:00 on the same day for all plants. The fourth fully expanded leaf was collected from each of the 30 plants using a razor blade, immediately placed in a humidified plastic bag, and transported to the laboratory. Each leaf was positioned inside the chamber with the cut petiole exposed, and pressure was gradually increased until xylem sap appeared at the cut surface. The balancing pressure was initially recorded in bar and converted to MPa using 1 bar = 0.1 MPa before statistical analysis and preparation of Figure 1. The converted values were reported with a negative sign to express leaf water potential.

SPAD values were measured using a chlorophyll meter (SPAD-502, Konica Minolta, Tokyo, Japan), beginning on day 4 and thereafter at 2-day intervals throughout the 45-day experimental period. Measurements were performed in the evening on the third fully expanded leaf of each plant. Three readings were obtained from each plant at every measurement date, resulting in five independent biological readings per treatment combination. Treatment values were expressed as the mean of the five plants. SPAD readings were used as a non-destructive indicator of relative leaf chlorophyll content and were not interpreted as direct measurements of photosynthetic rate.

Detached-leaf water loss was evaluated gravimetrically at the end of the experimental period. The third fully expanded leaf was excised from each of the 30 plants in the morning, immediately placed in a humidified plastic bag, and transported to the Tropical Horticultural Science Laboratory. Each leaf was weighed individually to obtain its initial fresh weight and then placed on a tray with the cut petiole left unsealed. All leaves were evaluated on the same day under a controlled temperature of 25 °C and RH of 63%. Leaves were reweighed at 10 min intervals for 60 min, resulting in five independent biological replicates per treatment combination. Percentage water loss at each measurement time was calculated relative to the initial fresh weight.

Leaf samples for metabolite analysis were collected from all 30 plants. The third fully expanded leaf was collected from each plant in the morning, and each plant was analyzed as an independent biological replicate. Metabolite extraction, derivatization, and GC–MS analysis were performed according to previously published procedures [22,23], with the specific conditions described below.

Each plant was processed as an independent biological replicate, and no samples were pooled. Immediately after collection, the leaves were transported to the Tropical Horticultural Science Laboratory and placed directly in a vacuum freeze-dryer (FDM-100, Tokyo Rikakikai Co., Ltd. [EYELA], Tokyo, Japan) for three days. Metabolite concentrations were expressed on a dry-weight basis.

A volume of 250 µL of methanol was added to each sample, followed by homogenization using a shaker (MM400, Retsch GmbH, Haan, Germany) at 27 Hz for 2 min. Samples were then centrifuged (C1008-B, Nichiryo Co., Ltd., Tokyo, Japan), and 250 µL of chloroform was added. The mixture was incubated in a thermomixer (Eppendorf Thermomixer PC2.0, Hamburg, Germany) at 37 °C and 1200 rpm for 3 min.

Subsequently, 50 µL of ribitol (internal standard) and 175 µL of ultrapure water were added, followed by vortex mixing (Vortex Genie 2, Scientific Industries Inc., Bohemia, NY, USA). Samples were centrifuged (MX-307, Tomy Seiko Co., Ltd., Tokyo, Japan), and the supernatant was collected and stored at −80 °C (MDF-C8V, Sanyo Electric Co., Ltd., Osaka, Japan) until analysis.

Prior to GC–MS analysis, samples were thawed at room temperature, and 80 µL of each extract was transferred into new 1.5 mL tubes. Solvent evaporation was performed using a centrifugal evaporator (Tokyo Rikakikai Co. Ltd. (EYELA), Tokyo, Japan) for 2 h, followed by freeze-drying at −40 °C overnight.

Derivatization was carried out by adding 40 µL of methoxyamine hydrochloride (20 mg mL^−1^ in pyridine) to each sample and incubating at 37 °C for 90 min at 1200 rpm. Subsequently, 50 µL of *N*-methyl-*N*-(trimethylsilyl)trifluoroacetamide (MSTFA) was added, followed by incubation at 37 °C for 30 min.

After derivatization, 50 µL of each sample was transferred into GC–MS vials. Metabolite profiling was performed using a gas chromatograph–mass spectrometer (GC-2010, Shimadzu Corporation, Kyoto, Japan). A total of 53 metabolites were detected and quantified. In the GC–MS output, GABA was identified as its trimethylsilyl derivative, 4-aminobutyric acid-3TMS. Therefore, this metabolite is referred to as γ-aminobutyric acid (GABA) throughout the manuscript. Selected metabolites were chosen based on their biological relevance and to represent several biochemical classes, including soluble sugars, sugar alcohols, amino acids and related nitrogenous compounds, and organic acids.

### 4.5. Statistical Analysis

Each individual plant represented one independent biological replicate, with five replicates per treatment combination. For SPAD measurements, the three readings obtained from each plant on each measurement date were averaged before calculating treatment means, thereby maintaining the individual plant as the experimental unit. Quantitative results are presented as means ± standard error where applicable.

Statistical analyses were performed using InfoStat software, version 2020d. Leaf water potential, final detached-leaf water content at 60 min, and the concentration of each selected metabolite were analyzed using two-way analysis of variance. Salinity level, glycine betaine application, and their interaction were included as fixed effects. When significant treatment effects were detected, means were compared using Tukey’s test. Statistical significance was evaluated at *p* < 0.05.

The detached-leaf water-loss time course was analyzed using repeated-measures analysis of variance, with salinity and glycine betaine application as between-subject factors and measurement time as the within-subject factor. The model included the interactions among salinity, glycine betaine application, and time. The initial measurement at 0 min was excluded from the inferential analysis because leaf water content was normalized to 100% for all samples at that time. SPAD temporal patterns were evaluated descriptively, and no inferential comparisons were performed among treatments or measurement dates.

Principal component analysis was conducted using XLSTAT, version 2025, as an exploratory analysis of all 53 detected metabolites across the 30 individual biological samples. The Z-score heatmap was generated using 15 selected metabolites standardized across all biological samples according to z = (x − μ)/s, where x is the individual metabolite value, μ is the mean, and s is the sample standard deviation. Principal component analysis and heatmap visualization were used to examine overall treatment-associated patterns and were not used for inferential hypothesis testing.

### 4.6. Experiment Disclosure

Declaration of Generative AI Use: During the preparation of this manuscript, the authors used ScholarGPT (OpenAI) to assist with language refinement and structural organization. The tool was not used to generate experimental data, perform laboratory procedures, or independently conduct statistical analyses. All scientific claims, interpretations, references, and final wording were reviewed and revised by the authors, who take full responsibility for the content of the manuscript.

## 5. Conclusions

Salinity level affected both the physiological status and primary metabolism of *P. edulis* f. *flavicarpa*, with the clearest GB-associated responses observed under the higher-salinity treatment. Exogenous glycine betaine partially mitigated some effects of higher salinity, particularly by maintaining less negative leaf water potential and modifying the accumulation of several stress-associated metabolites. However, its effects were not consistent across all physiological traits, indicating that the response to GB depends on the intensity of salinity stress.

The combined physiological and metabolomic results indicate that, under higher salinity, GB application was associated with improved plant water status and coordinated differences in several primary metabolite pools. These findings support further investigation of glycine betaine as a potential tool for improving passion fruit responses to salinity, particularly under conditions where soil or irrigation-water salinity limits crop productivity. Additional studies evaluating photosynthetic performance, oxidative stress markers, enzyme activities, gene expression, and longer-term plant responses are needed to clarify the mechanisms and agronomic relevance of the observed GB-associated responses.

## Figures and Tables

**Table 1:** Concentrations of selected metabolites in passion fruit seedlings under salinity and glycine betaine treatments. “S0” represents control salinity (no NaCl added), “S1” indicates lower salinity (1.6 dS m^−1^), and “S2” denotes higher salinity (3.2 dS m^−1^). “GB0” corresponds to no glycine betaine application, whereas “GB1” signifies glycine betaine application. Within each metabolite, means sharing at least one lowercase letter are not significantly different according to Tukey’s test at the 5% probability level. Values are presented as mean ± standard error (SE) (*n* = 5).

			Metabolite Concentration (μmol/g Dry Weight)			
		Proline	Glycine	Glucose	Fructose	Sucrose
Treatments	S0GB0	0.04 ± 0.004 b	0.06 ± 0.003 ab	3.66 ± 0.33 b	1.93 ± 0.71 a	10.01 ± 0.41 b
	S0GB1	0.04 ± 0.004 b	0.06 ± 0.007 ab	3.96 ± 0.5 b	1.31 ± 0.24 ab	9.28 ± 0.83 b
	S1GB0	0.02 ± 0.006 b	0.03 ± 0.01 cd	3.47 ± 0.48 b	0.13 ± 0.01 b	7.41 ± 1.51 bc
	S1GB1	0.14 ± 0.01 b	0.02 ± 0.0002 d	3.27 ± 0.41 b	0.43 ± 0.04 b	5.09 ± 0.09 cd
	S2GB0	0.35 ± 0.04 b	0.05 ± 0.001 bc	3.20 ± 0.06 b	1.00 ± 0.19 ab	3.92 ± 0.14 d
	S2GB1	1.77 ± 0.24 a	0.08 ± 0.001 a	7.31 ± 1.57 a	0.62 ± 0.11 ab	13.55 ± 0.51 a
		Malic Acid	Citric Acid	Succinic Acid	Glutamic Acid	γ-aminobutyric acid (GABA)
	S0GB0	3.63 ± 0.33 a	0.91 ± 0.15 b	0.27 ± 0.01 bc	0.02 ± 0.004 ab	1.50 ± 0.05 a
	S0GB1	3.43 ± 0.26 a	0.86 ± 0.04 bc	0.35 ± 0.04 b	0.03 ± 0.003 a	1.24 ± 0.12 a
	S1GB0	1.58 ± 0.26 b	0.85 ± 0.2 bc	0.20 ± 0.05 c	0.02 ± 0.008 ab	0.79 ± 0.17 b
	S1GB1	0.91 ± 0.03 b	0.40 ± 0.01 c	0.16 ± 0.002 c	0.003 ± 0.0003 b	0.55 ± 0.004 b
	S2GB0	0.97 ± 0.03 b	0.64 ± 0.009 bc	0.32 ± 0.006 b	0.01 ± 0.0002 ab	0.64 ± 0.007 b
	S2GB1	4.12 ± 0.11 a	1.92 ± 0.1 a	0.56 ± 0.01 a	0.02 ± 0.004 ab	1.63 ± 0.03 a

## Data Availability

The physiological and metabolomic data supporting the findings of this study are available from the corresponding author upon reasonable request.

## Abbreviations

The following abbreviations are used in this manuscript:

ANOVA	Analysis of variance
EC	Electrical conductivity
F1	First principal component axis
F2	Second principal component axis
GABA	γ-Aminobutyric acid
GB	Glycine betaine
GB0	No glycine betaine application
GB1	Glycine betaine application at 100 mM
GC–MS	Gas chromatography–mass spectrometry
MSTFA	*N*-Methyl-*N*-(trimethylsilyl)trifluoroacetamide
NaCl	Sodium chloride
PCA	Principal component analysis
PC1	First principal component
PC2	Second principal component
ROS	Reactive oxygen species
S0	Control salinity treatment, with no NaCl added
S1	Lower salinity treatment at 1.6 dS m^−1^
S2	Higher salinity treatment at 3.2 dS m^−1^
SE	Standard error
SPAD	Soil–Plant Analysis Development
TCA	Tricarboxylic acid
